# Protective Effects of Hesperidin (Citrus Flavonone) on High Glucose Induced Oxidative Stress and Apoptosis in a Cellular Model for Diabetic Retinopathy

**DOI:** 10.3390/nu9121312

**Published:** 2017-12-02

**Authors:** Wayne Young Liu, Shorong-Shii Liou, Tang-Yao Hong, I-Min Liu

**Affiliations:** 1 Department of Urology, Jen-Ai Hospital, Taichung City 41625, Taiwan; waynedoctor@gmail.com; 2 Center for Basic Medical Science, College of Health Science, Central Taiwan University of Science and Technology, Taichung City 40601, Taiwan; 3 Department of Pharmacy and Master Program, College of Pharmacy and Health Care, Tajen University, Pingtung County 90741, Taiwan; ssliou@tajen.edu.tw; 4 Department of Biotechnology, Collage of Pharmacy and Health Care, Tajen University, Pingtung County 90741, Taiwan; tyhong@tajen.edu.tw

**Keywords:** hesperidin, high glucose, retinal ganglial cells, apoptosis

## Abstract

The aim of this study was to investigate the protective effects and mechanisms of hesperidin, a plant based active flavanone found in citrus fruits, under the oxidative stress and apoptosis induced by high levels of glucose in retinal ganglial cells (RGCs). RGC-5 cells were pretreated with hesperidin (12.5, 25, or 50 μmol/L) for 6 h followed by exposure to high (33.3 mmol/L) d-glucose for 48 h. The 3-(4,5-dimethylthiazol-2-yl)-2,5-diphenyltetrazolium bromide (MTT) assay was adopted to evaluate cell viability. Mitochondrial function was estimated by measuring the mitochondrial membrane potential (ΔΨm). A fluorescent probe was employed to evaluate the intercellular production of reactive oxygen species (ROS). Colorimetric assay kits were used to evaluate lipid peroxidation, antioxidant enzyme activities, and protein carbonyls formation. The expression of apoptosis-related proteins and mitogen-activated protein kinase (MAPK) were measured with Western blotting. Hesperidin inhibited high glucose-mediated cell loss and restored mitochondrial function including a reversion of ΔΨm loss and cytochrome c release. Treated with hesperidin, high glucose-induced increase in ROS, malondialdehyde, and protein carbonyl levels were blocked in RGC-5 cells. Hesperidin was found to elevate the activities of superoxide dismutase, catalase, glutathione peroxidase, and to recover glutathione levels. Hesperidin inhibited high glucose-induced cell apoptosis by attenuating the downregulation of caspase-9, caspase-3, and Bax/Bcl-2. Furthermore, the phosphorylation of c-Jun N-terminal kinases (JNK) and p38 MAPK triggered by high glucose were attenuated in RGC-5 cells after their incubation with hesperdin. We concluded that hesperidin may protect RGC-5 cells from high glucose-induced injury since it owns the properties of antioxidant action and blocks mitochondria-mediated apoptosis.

## 1. Introduction

Diabetes mellitus (DM) and its complications are becoming an increasingly serious global public health issue, with a great increase in the number of patients worldwide. Diabetic retinopathy (DR) is one of the major and most common microvascular complications of DM, and is the most important cause of blindness occurring in adults [1]. Studies have shown that the primary pathology in DR is related to the dysfunction and death of retinal ganglion cells (RGCs), the only efferent neurons that transmit visual information from the retina to the visual center [2,3]. A high glucose level may alter the structure and function of RGCs, leading to visual field defects, and ultimately blindness [3]. Continuous hyperglycemia during diabetes incurs a redox imbalance caused by the overproduction of reactive oxygen species (ROS) [4]. Sustaining high levels of ROS damages mitochondrion, which ultimately leads to the apoptosis of RGCs [5]. Several biochemical changes have been proposed as essential events that commit a cell to undergo mitochondria-associated apoptosis, which can be mediated by activating pro-apoptotic Bcl-2 family proteins, increasing oxidative stress, initiating a caspase cascade, loss of mitochondrial membrane potential (ΔΨm), and cytochrome c redistribution [6,7]. Hence, the regulation of hyperglycemia-induced oxidative stress and associated cell death in RGCs would be essential prevention and treatment strategies for DR.

Epidemiological studies have shown that a large number of phytochemicals in fruits and vegetables might protect the human body against ROS-related pathologies [8]. Therefore, phytochemicals extracted from plant sources have drawn increasing attention since they are potential agents in the prevention and treatment of oxidative stress-related diseases [9]. Hesperidin (hesperetin 7-rutinoside) is an inexpensive flavone glycoside rich in citrus fruits and isolated from the ordinary orange *Citrus aurantium* L. and other plants of the family *Rutaceae* [10]. Hesperidin improves the health of capillaries by reducing the capillary permeability [11]. Evidence presented in numerous in vitro and in vivo studies has shown that hesperidin possesses analgesic, anti-hypertensive, and diuretic activity; is hypolipidemic; and exhibits obvious anti-inflammatory activity [12]. Hesperidin inducing apoptosis has been reported in various cancer cells including colon, liver, and mammary cancer cells [13,14,15]. On the contrary, this flavanone displays anti-apoptotic properties in neuroblastoma and human keratinocyte cell lines [16,17]. Another potential therapeutic application of hesperidin is in ocular diseases. It has been demonstrated that hesperidin possesses the ability to attenuate hyperglycemia-mediated oxidative stress and pro-inflammatory cytokine production in high fat fed/streptozotocin-induced type 2 diabetic rats [18]. Similar results have been documented where hesperidin possesses the ability to attenuate retina abnormalities via anti-angiogenic, anti-inflammatory, and antioxidative effects, as well as the inhibitory effect on the polyol pathway and advanced glycosylation end product accumulation in diabetic rats [19]. Based on different studies, hesperidin is considered as a potential therapeutic compound for DM and related complications. Thus, it is valuable to clarify whether the protective effects of hesperidin on retinal abnormalities under high glucose is involved in the amelioration of oxidative stress and associated cell death.

However, the role of hesperidin on the amelioration of mitochondrial dysfunction and death of RGCs caused by high glucose has not clarified thus far. To understand the influence of hyperglycemia on the pathogenesis of diabetes, a common model is to expose high glucose concentrations in vitro [20]. Thus, we elucidated the retina-protective effect of hesperidin on RGC-5 model of DR in vitro under high-glucose conditions by analyzing its effect on high glucose mediated oxidative stress generation, mitochondrial dysfunction, and apoptosis. 

## 2. Materials and Methods

### 2.1. Cell Culture

The retinal ganglion cell 5 (RGC-5) cells, purchased from American Type Culture Collection (Manassas, VA, USA), have been previously characterized as expressing ganglion cell markers and exhibiting ganglion cell-like behavior in culture [21]. RGC-5 cells were cultured in Dulbecco’s modified Eagle’s medium (DMEM: l-glutamine, 110 mg/L sodium bicarbonate and 1 g/L d-glucose) containing 10% fetal bovine serum (Sigma-Aldrich, St. Louis, MO, USA), and penicillin-streptomycin (100 U/mL and 100 μg/mL, Sigma-Aldrich, St. Louis, MO, USA) in a humidified incubator with 5% CO_2_ at 37 °C. The doubling time of these cells was approximately 20 h under these conditions. The medium was changed every other day, and the cells were passaged at a ratio of approximately 1:8 every 2–3 days. RGC-5 cells of passages 10–20 were used in these studies.

### 2.2. High-Glucose Stimulation

Cells were seeded at a density of 2 × 10^6^ cells/well in 6-well plates. Upon confluence, cultures were passaged by dissociation in 0.05% (*w*/*v*) trypsin (Sigma-Aldrich) in phosphate-buffered saline (PBS) pH 7.4. For high-glucose-induced functional studies, cells were maintained in fresh medium containing 1% fetal bovine serum for 2 h prior to use in the experiments. Later, cells were pretreated with hesperidin (purity ≥ 97%; Cat. No. 50162; Sigma-Aldrich) at a variety of concentrations (12.5, 25, or 50 μmol/L), or vehicle for 6 h followed by further exposure to normal (5.5 mmol/L), or high (33.3 mmol/L) d-glucose for 48 h without medium change. The dosage regimen was selected based on the previous reports demonstrating that hesperidin protected human keratinocytes from ultraviolet B radiation-induced apoptosis and was potentially effective in decreasing vascular formation in endothelial cells [17,22]; never RGCs be exposed to these concentrations. Hesperidin powder was dissolved in dimethyl sulfoxide (DMSO) (purity ≥ 99%; Cat. No. W387520; Sigma-Aldrich) to create a stock at a concentration of 10 mg/mL, subsequently diluted in culture medium to the corresponding concentration for the following experiments. DMSO was used as the vehicle control in the experiments. The final DMSO concentration did not exceed 0.1% (*v*/*v*), a concentration that did not affect cell viability. Afterwards, cell viability, antioxidant enzyme activities, reactive oxygen species (ROS) generation, and several apoptotic indexes were assessed. Triple wells were evaluated in each experiment, and each experiment was performed at least five times independently.

### 2.3. Cell Survival Assay

The viability of RGC-5 cells exposed to normal or high glucose plus either hesperidin was determined by a 3-(4,5-dimethylthiazol-2-yl)-2,5-diphenyltetrazolium bromide (MTT; Sigma-Aldrich) assay, as previously described with modifications in Reference [23]. Typically, 10,000 cells suspended in 100 μL of media were incubated with 10 μL of MTT reagent for approximately 3 h, followed by the addition of a detergent solution to lyse the cells and solubilize the colored crystals. The absorbance of the samples was measured at 570 nm using a microplate reader (MTP-800; CORONA, Tokyo, Japan). The cell viability in the control medium without any treatment was represented as 100% and the cell viability percentage of each well was calculated. Each experiment was conducted in three wells and was duplicated at least five times.

### 2.4. Detection of Intracellular Reactive Oxygen Species (ROS)

The generation of intracellular ROS was assessed using 2′,7′-dichlorodihydrofluorescein diacetate (DCFDA), a non-fluorescent probe, which upon oxidation by ROS and peroxides is converted to the highly fluorescent derivative dichlorofluorescin (DCF) [24]. We used a DCFDA cellular ROS detection assay kit (Abcam, Cambridge, MA, USA) as per the manufacturer’s instructions. In brief, an aliquot of the isolated cells (8 × 10^6^ cells/mL) was made up to a final volume of 2 mL in normal PBS (pH 7.4). Then, a 1 mL aliquot of cells was taken to which 100 µL DCFDA (10 µmol/L) was added and incubated at 37 °C for 30 min and washed twice with PBS. Cells were solubilized in Triton-X100 1% (*v*/*v*) in distilled water. The cells were then examined under a fluorescence microscope (C1-T-SM; Nikon, Tokyo, Japan). The percentage of fluorescence-positive cells was measured at an excitation wavelength of 488 nm and an emission wavelength of 535 nm by a fluorescence spectrophotometer (F-2500; Hitachi, Tokyo, Japan).

### 2.5. Estimation of Lipid Peroxidation and Protein Carbonyl Content

Lipid peroxidation was studied by measuring the amount of malondialdehyde (MDA) in the cell homogenates using the lipid peroxidation assay kit (Abcam) as per the manufacturer’s instructions. Briefly 1 mmol/L ethylene diamine tetraacetic acid (Sigma, St. Louis, MO, USA) was added to a 0.5 mL cell lysate (6 × 10^6^ cells/mL) and was mixed with 1 mL cold 15% (*w*/*v*) thiobarbituric acid to precipitate proteins. The supernatant was treated with 1 mL 0.5% (*w*/*v*) TBA in a boiling water bath for 15 min. After cooling, the absorbance was read at 535 nm, and the concentration of the thiobarbituric acid reactive substance was calculated by using malondialdehyde as a standard [25]. Results were expressed as mmol thiobarbituric acid reactive substances/mg of protein. Protein carbonyl contents in the cell homogenates were determined with a protein carbonyl content assay kit (Abcam) according to the manufacturer’s procedures. The assessment of carbonyl formation was done based on the formation of protein hydrazone by reaction with 2,4-dinitrophenylhydrazine [26]. The absorbance was measured at 370 nm. The carbonyl content was calculated using the absorption coefficient of 22,000/M/cm. Results were expressed as mmol/mg of protein. The protein content of the sample was assessed using the Bio-Rad Protein Assay kit (Bio-Rad Laboratories Inc., Hercules, CA, USA) as per the manufacturer’s instructions.

### 2.6. Assay of Antioxidant Enzymes

Cells were seeded at a density of 2 × 10^6^ cells/well in 6-well plates. The activities of antioxidant enzymes were assayed in cells treated with test samples as following.

Superoxide dismutase (SOD) activity was assayed using a SOD activity colorimetric assay kit (BioVision Inc., San Francisco, CA, USA) which utilizes a highly water soluble tetrazolium salt, 2-(4-indophenyl)-3-(4-nitrophenyl)-5-(2,4-disulfophenyl)-2H-tetrazolium, monosodium salt, that produces a water soluble dye on reduction with O_2_^−^. The absorbance was measured at 450 nm. The rate of inhibition of the activity of xanthine oxidase by SOD was measured and expressed as the enzyme required for a 50% inhibition of xanthine oxidase activity per minute per milligram protein.

A glutathione peroxidase (GPx) activity assay was performed by using a GPx colorimetric assay kit (BioVision Inc.) following the manufacturer’s instructions. The kit is based on the oxidation of glutathione (GSH) to oxidized glutathione (GSSG) catalyzed by GPx, which is then coupled to the recycling of GSSG back to GSH utilizing the glutathione reductase and nicotinamide adenine dinucleotide phosphate. Cell extracts were prepared with a glutathione peroxidase assay buffer with 50 mmol/L Tris HCl, adjusted with HCl to pH 7.0, and contained 0.5 mmol/L EDTA, and used in the assay using the protocol described in the assay kit. The rate of nicotinamide adenine dinucleotide phosphate oxidation by H_2_O_2_ was monitored at 340 nm and GPx activity was expressed in unit/mg protein.

The catalase (CAT) activity in cells treated with test samples was assayed using the catalase assay kit by Cayman Chemical (Ann Arbor, MI, USA). The assay was based on the reaction of methanol and the enzyme in the presence of an optimal concentration of H_2_O_2_. The formaldehyde produced was measured colorimetrically at 540 nm using 4-amino-3-hydrazino-5-mercapto-1,2,4-triazole (Purpald; Sigma-Aldrich, St. Louis, MO, USA) as the chromogen. CAT activity was expressed in unit/mg protein.

The levels of glutathione (GSH) in the cells were determined by using a glutathione colorimetric assay kit (BioVision Inc.). Briefly, cells were washed and taken to determine GSH levels by following the procedures provided by the manufacturer. The sample was first deproteinized with the 5% 5-sulfosalicylic acid solution. The GSH content of the sample was then assayed using a kinetic assay where the catalytic amounts of glutathione caused a continuous reduction of 5,5′-dithiobis-(2-nitrobenzoic) acid to 2-nitro-5-thiobenzoic acid (TNB). The oxidized GSH formed was recycled by the GSH reductase and NADPH. TNB was assayed colorimetrically at 412 nm. The GSH level was expressed in nmol/mg protein.

### 2.7. Measurement of Mitochondrial Membrane Potential (ΔΨm)

A mitochondrial membrane potential assay kit containing JC-1 (Cat. No. ab113850; Abcam) was used to measure the ΔΨm in RGC-5 cells. Briefly, 1 × 10^4^ cells were laid in a 96-well plate and incubated with 20 µmol/L JC-1 in growth medium at 37 °C for 30 min. Monomeric JC-1 green fluorescence emission (530 nm) and aggregate JC-1 red fluorescence emission (590 nm) were determined using a fluorescence spectrophotometer (F-2500; Hitachi). The ΔΨm in each group was calculated as the fluorescence ratio of red to green. This value was adjusted by subtracting the value obtained from the carbonyl cyanide 3-chlorophenylhydrazone incubation and expressed as a percentage of the vehicle control.

### 2.8. Measurement of Cytochrome C Release

A mitochondrial fractionation kit (Active Motif Inc., Carlsbad, CA, USA) was utilized to isolate the mitochondrial and cytosolic fractions from cells under the manufacturer’s instructions. Briefly, the cells (8 × 10^6^ cells/mL) were scraped and spun twice at 600× *g* for 5 min. Ice-cold 1× cytosolic buffer was added into the cell pellet, which was then resuspended and incubated on ice for 15 min. Cells were homogenized and the lysate was spun twice at 800× *g* for 20 min. The resultant supernatant was centrifuged at 10,000× *g* for 20 min to pellet the mitochondria. The mitochondrial fraction of protein was obtained from the mitochondrial pellet lysed by adding complete mitochondria buffer, and incubating on ice for 15 min. Meanwhile, the supernatant was centrifuged at 16,000× *g* for 25 min to obtain the cytosolic fraction. Protein concentration was determined by the Bradford assay. After isolating the mitochondria and cytosolic fraction, the cytochrome c enzyme-linked immunosorbent assay kit (Abcam) was used to measure the level of cytochrome c as per the manufacturer’s instructions.

### 2.9. Western Blot Analysis

Proteins were prepared from the cytosolic and mitochondrial samples or whole-cell lysis of cells (8 × 10^6^ cells/mL). The mitochondrial fractionation kit (Cat. No. 40015; Active Motif Inc., Carlsbad, CA, USA) was used to isolate cytosolic and mitochondrial fractions from cells according to the manufacturer’s instructions. In brief, cells were harvested and centrifuged at 800× *g* at 4 °C for 10 min, and the cell suspension was then placed into a glass homogenizer and homogenized for 30 strokes by using a tight pestle on ice. Homogenates were centrifuged at 800× *g* at 4 °C for 10 min to collect the supernatant, which was further centrifuged at 10,000× *g* at 4 °C for 20 min to obtain the cytosol (supernatant) and mitochondria (deposition) fraction. Protein concentrations were determined using the Bradford protein assay (Bio-Rad Laboratories). Equal amounts of protein (30 μg/lane) were resolved by electrophoresis for Western blot analysis. Protein was separated by electrophoresis on 10% sodium dodecyl sulfate-polyacrylamide gel electrophoresis and transferred electrophoretically to polyvinylidene difluoride membranes. Membranes were blocked with 5% non-fat dry milk in Tris-buffered saline Tween (20 mmol/L Tris, pH 7.6, 137 mmol/L NaCl, and 0.1% Tween 20) for 3 h at room temperature, accompanied by an overnight incubation at 4 °C with primary antibodies against cytochrome c (Cat. No. 13156; Santa Cruz Biotechnology Inc., Santa Cruz, CA, USA), cleaved caspase-3 (Cat. No. 9661; Cell Signaling Technology, Beverly, CA, USA), cleaved caspase-9 (Cat. No. 9501; Cell Signaling Technology), Bcl-2 (Cat. No. sc-492; Santa Cruz Biotechnology Inc.), Bax (Cat. No. sc-526; Santa Cruz Biotechnology Inc.), c-Jun N-terminal kinases (JNK) (Cat. No. sc-137020; Santa Cruz Biotechnology Inc.), p-JNK (Thr 183/Tyr 185) (Cat. No. sc-6254; Santa Cruz Biotechnology Inc.), p38 (Cat. No. 9212; Cell Signaling Technology), or p-p38 (Thr180/Tyr182) (Cat. No. 9211; Cell Signaling Technology). The β-Actin antibody (Cat. No. sc-130656; Santa Cruz Biotechnology Inc.) was used as an internal control to exclude the putative contamination of mitochondrial fraction by cytosolic proteins. The cytochrome c oxidase subunit IV isoform 1 (CoxIV) antibody (Cat. No. 4850; Cell Signaling Technology) was used as a mitochondrial loading control. All antibodies were used at a dilution of 1:1000. After being washed three times with Tris-buffered saline Tween 20 (TBST), incubation with the appropriate horseradish peroxidase-conjugated secondary antibodies was performed for one hour at room temperature. After three additional TBST washes, the immunoreactive bands were visualized by enhanced chemiluminescence (Amersham Biosciences, Buckinghamshire, UK) as per the manufacturer’s instructions. Band densities were calculated with ATTO Densitograph Software (ATTO Corporation, Tokyo, Japan) and quantified as the ratio to β-actin or CoxIV.

The mean value for samples from the vehicle treated cells cultured under normal glucose on each immunoblot, expressed in densitometry units, was adjusted to a value of 1.0. All experimental sample values were then expressed relative to this adjusted mean value. All determinations were performed in triplicate, and each experiment was repeated at least five times.

### 2.10. Statistical Analysis

Data were expressed as the mean ± standard deviation (SD). Statistical analysis and graphics were made with a SigmaPlot 12.3 program (version 2016, Systat Software Inc., San Jose, CA, USA). Statistical analysis was conducted with one-way analysis of variance (ANOVA). Dunnett range post-hoc comparisons were employed to determine the source of significant differences where appropriate. A *p*-value < 0.05 was considered statistically significant.

## 3. Results

### 3.1. Effect of Hesperidin on High Glucose-Induced Cell Death

**Figure 1:**
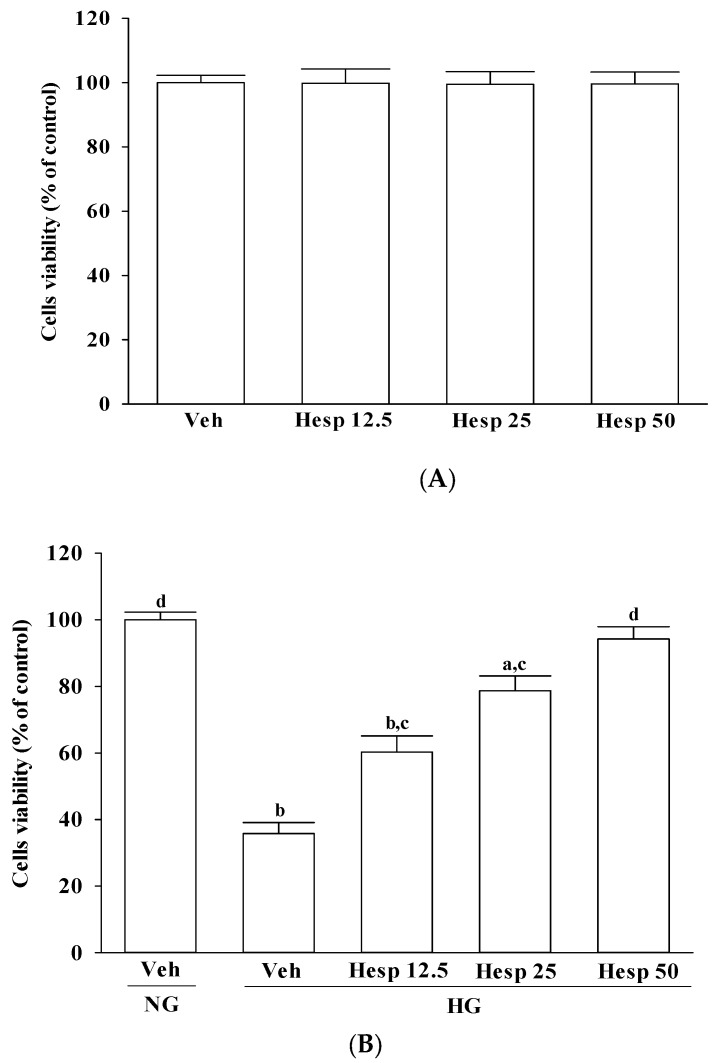
Cytotoxicity of hesperidin to RGC-5 cells. The RGC-5 cells were cultured with normal (NG) or high glucose (HG) plus hesperidin at concentrations of 12.5 (Hesp 12.5), 25 (Hesp 25), or 50 (Hesp 50) μmol/L for 48 h. (**A**) Effects of treatments on cell viability in RGC-5 cells cultured with normal glucose concentration; (**B**) Effects of treatments on cell viability in RGC-5 cells cultured with high glucose concentration. The cell viability was determined by the 3-(4,5-dimethylthiazol-2-yl)-2,5-diphenyltetrazolium bromide (MTT) assay. The results are presented as the mean ± standard deviation (SD) of five independent experiments (*n* = 5), each of which was performed in triplicate. ^a^ *p* < 0.05 and ^b^ *p* < 0.01 when compared with the normal-glucose vehicle (Veh)-treated group. ^c^ *p* < 0.05 and ^d^ *p* < 0.01 when compared with the high-glucose vehicle-treated group.

The incubation of normal glucose-cultured RGC-5 cells with hesperidin at 12.5, 25, or 50 μmol/L for 48 h had little or no effect on cytotoxicity (>90% viability remaining) (Figure 1A). The protective effects of hesperidin against high glucose-induced cell death in RGC-5 cells are shown in Figure 1B, where cell viability was about 30.2% in high-glucose cultured cells, whereas hesperidin recovered the cell death caused by high glucose in a concentration-dependent manner with almost 94.2% of the cells surviving at a 50 μmol/L.

### 3.2. Effects of Hesperidin on ROS Production, Lipid Peroxidation, Protein Carbonylation, and Antioxidant Enzymatic Activities in RGC-5 Cells under High Glucose Environment

**Figure 2:**
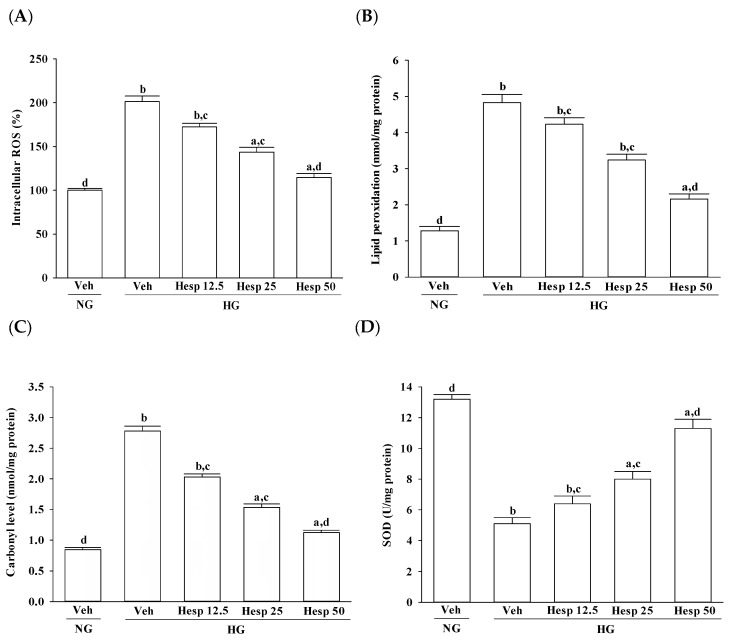

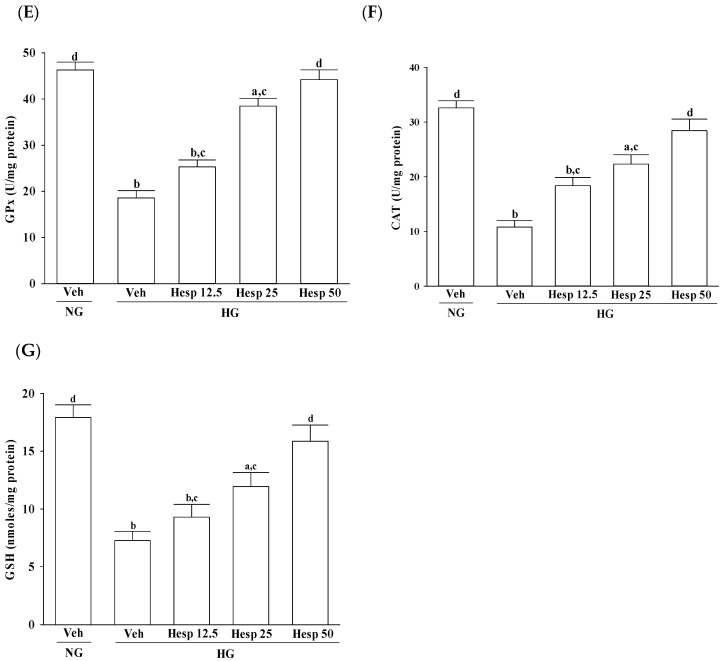
Effects of hesperidin on reactive oxygen species (ROS)production, lipid peroxidation, protein carbonylation, and endogenous antioxidant defense in RGC-5 cells following high-glucose challenge: (**A**) ROS levels; (**B**) lipid peroxidation levels; (**C**) total protein carbonyl content; and (**D**) levels of: superoxide dismutase (SOD); (**E**) glutathione peroxidase (GPx); (**F**) catalase (CAT); and (**G**) glutathione (GSH). The RGC-5 cells were cultured with normal (NG) or high glucose (HG) plus hesperidin at concentrations of 12.5 (Hesp 12.5), 25 (Hesp 25), or 50 μmol/L for 48 h. The experiments were performed in triplicate and data are presented as mean ± SD of five independent experiments (*n* = 5). ^a^ *p* < 0.05 and ^b^ *p* < 0.01 when compared with the normal-glucose vehicle (Veh)-treated group. ^c^ *p* < 0.05 and ^d^ *p* < 0.01 when compared with the high-glucose vehicle-treated group.

The intracellular levels of ROS, MDA, and protein carbonyl in RGC-5 cells under a high glucose environment were higher by about 2.1-, 3.8-, and 3.3-fold, respectively in high glucose cultured cells when compared to the normal-glucose vehicle-treated group (Figure 2A–C). The higher levels of ROS, MDA, and protein carbonyl in RGC-5 cells under high glucose were downregulated by hesperidin (50 μmol/L) treatment with a decrease of 42.9%, 55.3%, and 59.7%, respectively, relative to those observed in their vehicle-treated counterparts (Figure 2A–C).

In high glucose conditions, the activities of SOD, GPx, and CAT were decreased sharply in RGC-5 cells (Figure 2D–F). In high glucose-cultured RGC-5 cells with a concentration-dependent manner, hesperidin recovered the reduced activities of SOD, GPx, and CAT (Figure 2D–F). Compared with the normal-glucose group, the concentration of GSH was 40.5% lower in the high glucose cultured RGC-5 cells (Figure 2G). In their vehicle-treated counterpart group, hesperidin concentration (50 μM) upregulated GSH level to 2.2-fold of the levels (Figure 2G).

### 3.3. Effects of Hesperidin on the Mitochondrial Membrane Potential (ΔΨm) and Cytochrome C Release in RGC-5 Cells under High Glucose Conditions

**Figure 3:**
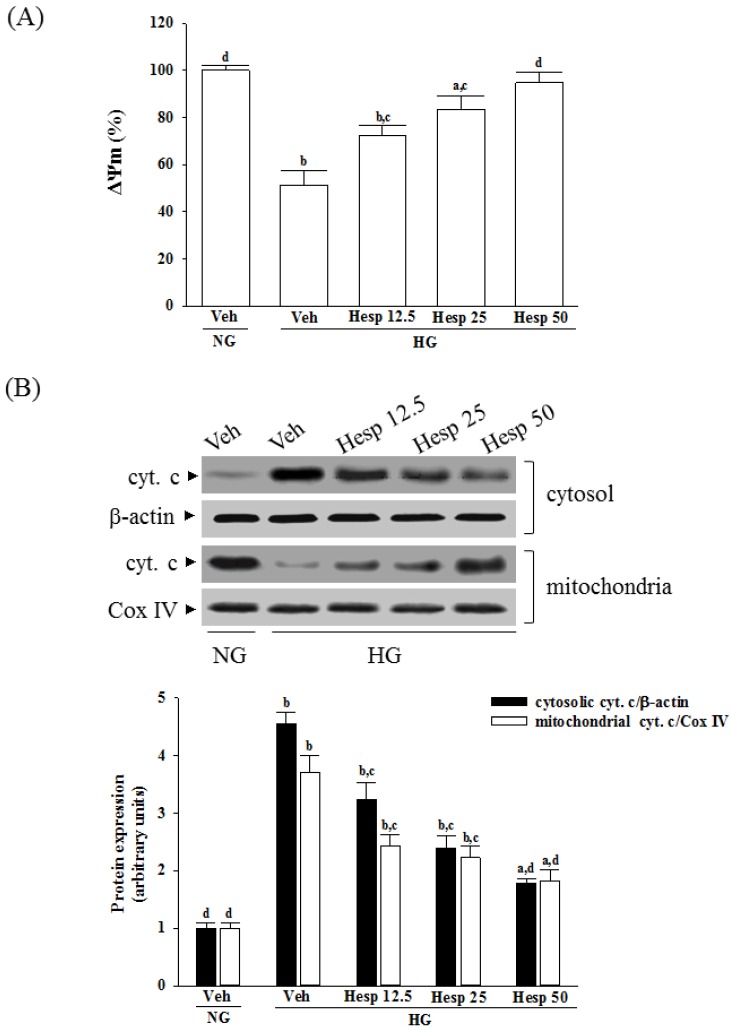
Effects of hesperidin on mitochondrial function in high glucose-treated RGC-5 cells. The RGC-5 cells were cultured with normal (NG) or high glucose (HG) plus hesperidin at concentrations of 12.5 (Hesp 12.5), 25 (Hesp 25), or 50 μmol/L (Hesp 50) for 48 h. (**A**) Effects of treatments on high glucose-induced reduction of mitochondrial membrane potential (ΔΨm); (**B**) Effects of treatments on high glucose-induced release of cytochrome c (cyt. c) from mitochondria. The experiments were performed in triplicate and data are presented as the mean ± SD of five independent experiments (*n* = 5). ^a^ *p* < 0.05 and ^b^ *p* < 0.01 when compared with the normal-glucose vehicle (Veh)-treated group. ^c^ *p* < 0.05 and ^d^ *p* < 0.01 when compared with the high-glucose vehicle-treated group.

The ΔΨm in RGC-5 cells exposed to high glucose was reduced to 51.3 ± 3.6% of that in the normal-glucose group (Figure 3A). Treatment of high glucose-cultured RGC-5 cells with hesperidin increased ΔΨm in a concentration-dependent manner (Figure 3A). 

It was shown that decreased mitochondrial concentrations of cytochrome c were followed by increased cytosolic concentrations in RGC-5 cells cultured under high glucose, while hesperidin inhibited the release of cytochrome c from mitochondria to cytoplasm in a concentration-dependent manner (Figure 3B).

### 3.4. Effects of Hesperidin on Protein Expression Related to Apoptosis in RGC-5 Cells under High Glucose Conditions

**Figure 4:**
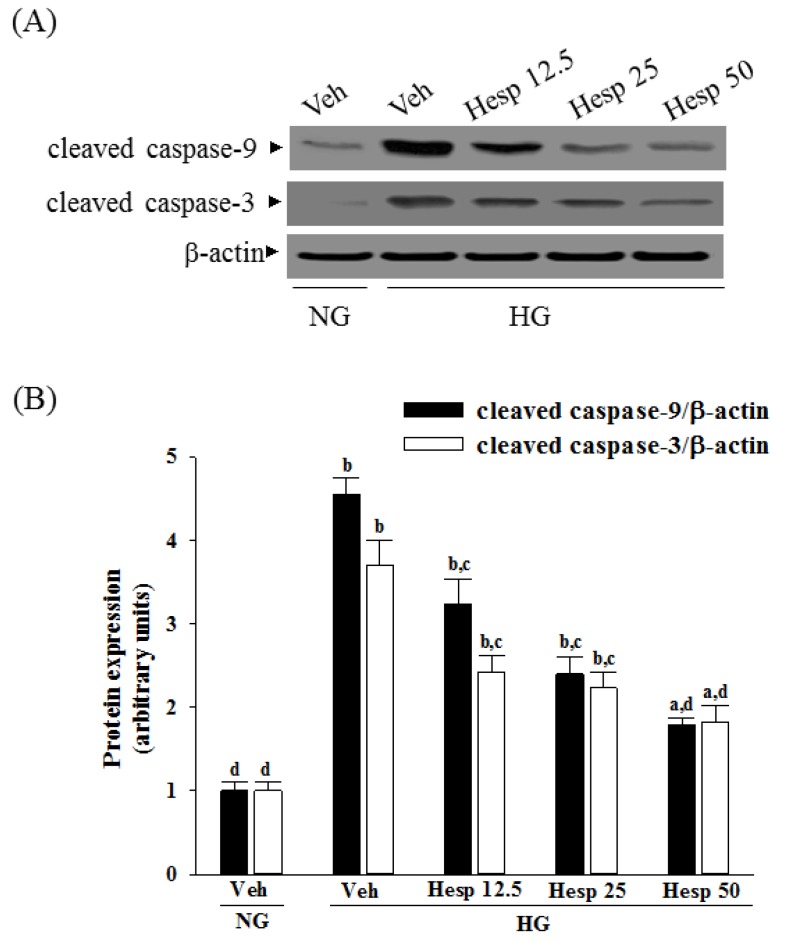

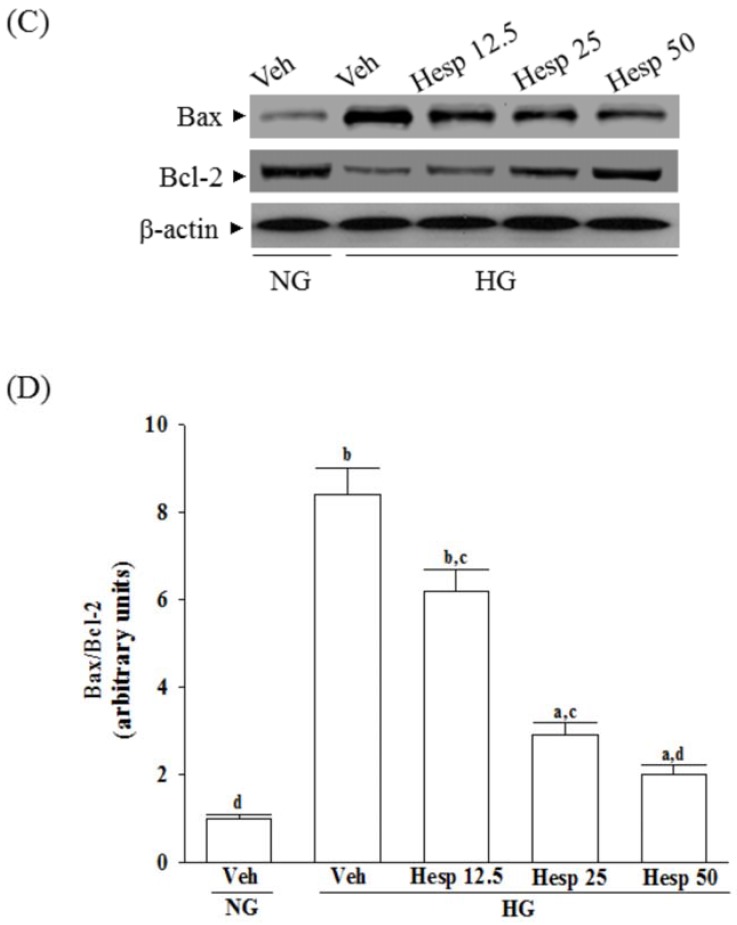
Effects of hesperidin on protein expression related to apoptosis in RGC-5 cells under high glucose conditions. The RGCs were cultured with normal (NG) or high glucose (HG) plus hesperidin at concentrations of 12.5 (Hesp 12.5), 25 (Hesp 25), or 50 μmol/L (Hesp 50) for 48 h. (**A**) Protein bands of cleaved caspase-9 and cleaved caspase-3 in RGC-5 cells detected by Western blotting; (**B**) Quantitative densitometric analysis of caspase-9 and cleaved caspase-3; (**C**) Protein bands of Bax and Bcl-2 in RGC-5 cells detected by Western blotting; (**D**) Changes of the ratios of Bax/Bcl-2 are displayed in the bottom panel. The results are presented as the mean ± SD of five independent experiments (*n* = 5), each of which was performed in triplicate. ^a^ *p* < 0.05 and ^b^ *p* < 0.01 when compared with the normal-glucose vehicle (Veh)-treated group. ^c^ *p* < 0.05 and ^d^ *p* < 0.01 when compared with the high-glucose vehicle-treated group.

High glucose caused a 4.6-fold and 3.7-fold increase in cleaved caspase-9 and cleaved caspase-3 protein expression in RGC-5 cells (Figure 4A,B). The protein expression of cleaved caspase-9 and cleaved caspase-3 in high glucose cultured RGC-5 cells was sharply decreased (58.3% and 50.9% reduction, respectively) by treatment with hesperidin (50 μmol/L) when compared to those of the vehicle-treated counterparts (Figure 4A,B).

High glucose greatly increased the Bax/Bcl-2 ratio in RGC-5 cells (by 8.4-fold relative to that seen in the normal-glucose vehicle-treated group; Figure 4C,D). This high glucose-induced up-regulation in the Bax/Bcl-2 ratio was reversed in the RGC-5 cells after treatment with 50 μmol/L hesperidin, with a 76.2% decrease when compared to that of their vehicle-treated counterpart group (Figure 4C,D).

### 3.5. Effects of Hesperidin on JNK and p38 Phosphorylation in RGC-5 Cells under High Glucose Conditions

**Figure 5:**
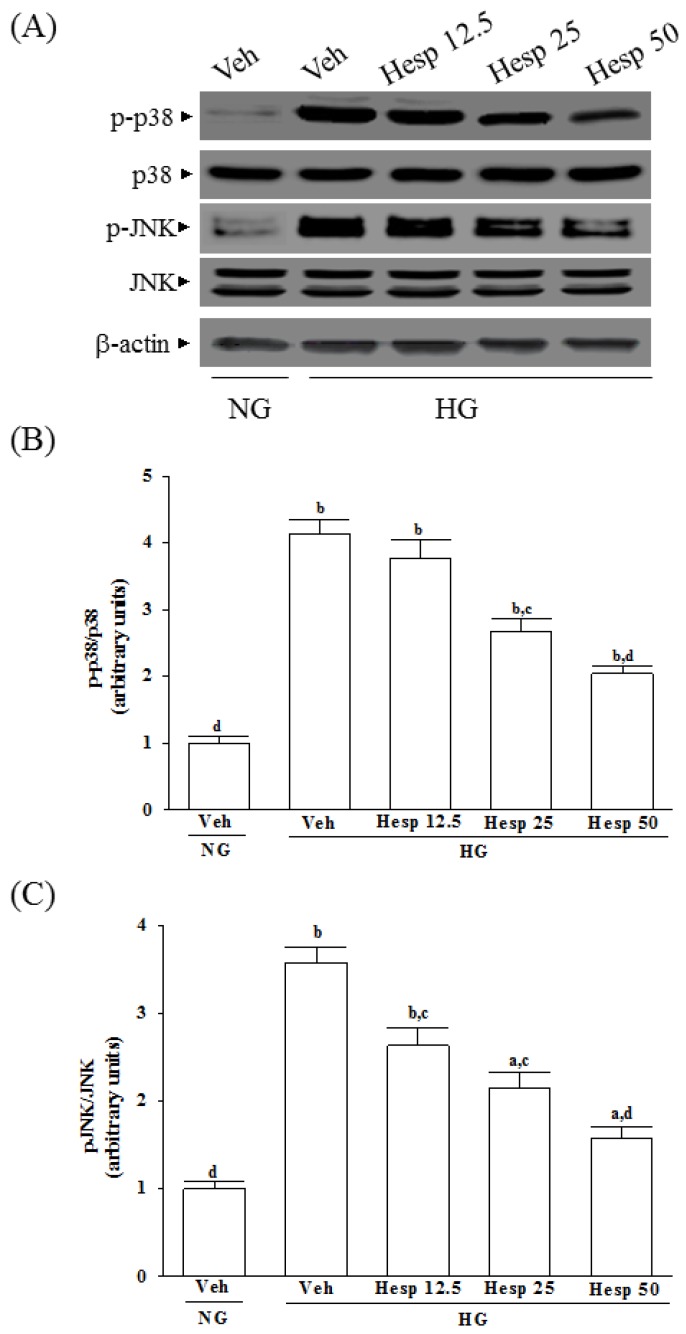
Effect of hesperidin on p38 and c-Jun N-terminal kinases (JNK) activation in high glucose cultured RGC-5 cells. (**A**) The photographs were representatives the Western blots for p-p38, p38, p-JNK, JNK and β-actin. Five western blots in each group were tested; (**B**) The ratio of p-p38/p38 was calculated; (**C**) The ratio of p-JNK/JNK was calculated. The results are presented as the mean ± SD of five independent experiments (*n* = 5), each of which was performed in triplicate. ^a^ *p* < 0.05 and ^b^ *p* < 0.01 when compared with the normal-glucose vehicle (Veh)-treated group. ^c^ *p* < 0.05 and ^d^ *p* < 0.01 when compared with the high-glucose vehicle-treated group.

The immunoblot results showed that the ratio of p-p38/p38 (Figure 5A,B) and p-pJNK/pJNK (Figure 5A,C) were 4.1- and 3.6-fold greater in the high glucose cultured RGC-5 cells than those in the normal-glucose vehicle-treated group, respectively. Compared to those in the normal-glucose vehicle-treated group, treatment of high glucose cultured RGC-5 cells with hesperidin (50 μmol/L) apparently downregulated the ratios of p-p38/p38 and p-pJNK/pJNK to 1.9- and 1.6-fold (Figure 5B,C). Whether in the presence of hesperidin or not, the expression of total p38 and JNK protein did not change in RGC-5 cells treated with high glucose (Figure 5A).

## 4. Discussion

The generation of ROS under high glucose stress conditions has been established in many cell types [27,28]. When the ROS level exceeds the capacity of the antioxidant defense system, ROS initiates chain reactions by oxidizing cellular macromolecules like lipids and proteins, which in turn interrupts cellular activities, ultimately causing apoptosis [7]. In this study, high glucose-mediated oxidant injury was utilized as an in vitro model to examine the protective effects of hesperidin in RGCs. In accordance with previous studies described in Reference [29], higher MDA and carbonyl contents in RGC-5 cells cultured under high glucose were observed. The increase in MDA level is associated with the oxidative damage of membrane lipids since ROS in cells are elevated [30]. Increased carbonyl levels are also involved with ROS-mediated damage of proteins [31]. From this observation, an increase in high glucose-induced oxidative damage of the cell membrane was suggested. Treatment with hesperidin resulted in a reduction of elevated ROS and MDA levels and at the same time the level of protein carbonyl became higher. These data showed that hesperidin has an antioxidant property that protects RGC-5 cells against high glucose-induced oxidative stress.

In the progression of diabetes, accumulated oxidative stress and depleted antioxidant defenses play a major part. SOD, CAT, and GPx are enzymes that ruin peroxides and have an impact on the antioxidant defenses of an organism [32]. SOD acts to dismutate superoxide radicals to hydrogen peroxides, which is then acted upon by GPx [33,34]. CAT catalyzes the reduction of hydrogen peroxides and protects the tissues from highly reactive hydroxyl radicals [35]. Furthermore, GSH acts as a cosubstrate for GPx activity and as a cofactor for many enzymes [36]. A decreased GSH content may predispose the cells to weaken their defense against the condition of oxidative stress during several degenerative disease conditions including diabetes [37]. Phytochemicals with antioxidant activity are known to abrogate oxidative stress [8]. Our data are in accordance with the hypothesis that during high glucose-induced stress to RGC-5 cells, hesperidin has a large influence on the control of ROS levels through upregulating both the enzymic and nonenzymic antioxidant defense systems. Consequently, it is expected that the mechanisms of hesperidin to protect against hyperglycemia-induced changes in RGC-5 cells and its capacity to counter oxidative stress will be studied in depth.

Although hesperidin could induce apoptosis through either an extrinsic or an intrinsic in several cancer cells [13,14,15], flavonoid can act as pro-apoptotic or anti-apoptotic agents depending on their concentration, the cell system, the type or stage of the degenerative process [16,17]. High glucose-induced oxidative stress mediated cell damage plays a significant role in the reduction of mitochondrial membrane potential, disruption of the mitochondrial membrane, and the release of cytochrome c into the cytosol, which in turn triggers caspase cascades and initiates the intrinsic apoptotic pathway [38]. Our results showed that high-glucose stimulation caused serious impairment of the ΔΨm and indeed fostered the release of cytochrome c, and the involvement of mitochondria in high glucose-mediated RGCs apoptosis was confirmed. It also showed that treatment of high glucose exposed RGC-5 cells with hesperidin could restore mitochondrial function by blocking the dissipation of ΔΨm and releasing cytochrome c into the cytosol. A strong inverse association between cell viability and apoptosis rates was observed. In fact, there was no significant cytotoxicity effect produced by hesperidin in the cell under these experimental conditions. At each concentration, hesperidin increased the survival rate of RGC in high glucose; in particular, the action of hesperidin was most obvious at 50 μmol/L. Data gained from the MTT assay in this study also suggested the direct neuroprotective action of hesperidin against high glucose-mediated mitochondrial dysfunction in RGC-5 cells as a model of DR. 

In mitochondria-dependent apoptosis, the released cytochrome c activates caspase-9 and sequentially activates the downstream effector caspase-3 [39]. Moreover, mitochondrial membrane potential is regulated by a specific category of proteins known as the Bcl-2 family proteins [40]. The anti-apoptotic Bcl-2 family proteins such as Bcl-2 are basically localized into mitochondrial outer membranes and can block the membrane permeabilization, whereas proapoptotic members of this family like Bax are usually translocated to the mitochondrial outer membrane from the cytosol, and facilitates the permeabilization of the mitochondrial membrane and release of cytochrome c [40]. As a result, because hesperidin blocks high glucose-induced elevation of Bax and decreases Bcl-2 in high glucose exposed RGC-5 cells, it inhibits enzymatic activity of caspase-9/3 and lowers the ratio of Bax/Bcl-2. These clarified that hesperidin protected RGC-5 cells from high glucose-induced cell apoptosis through mitochondrial-mediated pathway. 

It is known that the mitogen-activated protein kinases (MAPKs), a family of protein kinases, are related to cell growth and ROS-mediated death [41]. It also shows that antioxidants can prevent either ROS accumulation or MAPK activation under oxidative stress conditions [42]. Three major classes of MAPKs are extracellular-signal regulated kinases 1 and 2 (ERK1/2), p38 MAPK/stress-activated protein kinases, and c-Jun N-terminal kinases (JNKs) [43]. The p38 and JNK MAPK pathways are generally recognized as pro-apoptotic pathways in response to cellular stresses such as hypoxic, or oxidative stress, whereas ERKs respond to mitogens and growth factors that regulate cell proliferation and differentiation [43]. Therefore, regulating the activity of the MAPK signaling pathway, particularly the activation of p38 and JNK, is vital for protecting cells from ROS injury and cellular death [44]. In the present study, the phosphorylation degree of p38 and JNK in high glucose exposed RGC-5 cells were greatly decreased under hesperidin treatment. Our findings support a protective role of hesperidin in RGC exposed to a high glucose challenge through the inhibition of p38 and JNK activation.

Human experiments should be designed based upon prior animal experimentation, it is difficult to directly convert the concentration of hesperidin into human intake dose in our in vitro study. It has been demonstrated that hesperidin at the daily oral dosage of 100 and 200 mg/kg could attenuate retina and plasma abnormalities in diabetic rats [19]; this is approximately 16 to 32 mg/kg oral intake for an adult human. Thus, animal studies will be arranged in the future to find out the effective dose of hesperidin for protection the retinal ganglion cell from high glucose-induced damage in vivo; the results can be used as a reference for the dosage of human body.

## 5. Conclusions

The current results provide evidence that the protective effect of hesperidin on RGC-5 cells against high glucose-induced oxidative are associated with properties relating to the inhibition of oxygen free radicals and the oxidation of lipids and protein. It plays a role in the enforcement of endogenous antioxidant defense mechanisms and the protection of mitochondrial function by the modulating Bcl-2 family members, as well as by inhibiting caspases activation via a ROS-dependent p38 and JNK signaling pathway. The findings of this study shed light on the pharmacological application of hesperidin to prevent and offer therapy to the high-glucose-induced damage of retinal ganglion cells and provides the theoretical basis for further development of hesperidin for preventing blindness in patients with diabetes and retinal eye diseases.
